# Erythropoietin: Recent Developments in the Treatment of Spinal Cord Injury

**DOI:** 10.1155/2011/453179

**Published:** 2011-07-04

**Authors:** Stephana Carelli, Giovanni Marfia, Anna Maria Di Giulio, Giorgio Ghilardi, Alfredo Gorio

**Affiliations:** ^1^Laboratory of Pharmacology, Department of Medicine, Surgery and Dentistry, University of Milan, Polo H. San Paolo, Via A. di Rudinì 8, 20142 Milan, Italy; ^2^Clinical Pharmacology, IRCCS Humanitas, Via Manzoni 56, Rozzano, 20089 Milan, Italy; ^3^Cerebrovascular Unit, IRCCS, Istituto Neurologico C Besta, Via Celoria 11, 20133 Milan, Italy; ^4^Department of Medicine, Surgery and Dentistry, University of Milan, Polo H. San Paolo, Via A. di Rudinì 8, 20142 Milan, Italy

## Abstract

Erythropoietin (EPO), originally identified for its critical function in regulating production and survival of erythrocytes, is a member of the type 1 cytokine superfamily. Recent studies have shown that EPO has cytoprotective effects in a wide variety of cells and tissues. Here is presented the analysis of EPO effects on spinal cord injury (SCI), considering both animal experiments concerning to mechanisms of neurodegeneration in SCI and EPO as a neuroprotective agent, and some evidences coming from ongoing clinical trials. The evidences underling that EPO could be a promising therapeutic agent in a variety of neurological insults, including trauma, are mounting. In particular, it is highlighted that administration of EPO or other recently generated EPO analogues such as asialo-EPO and carbamylated-EPO demonstrate interesting preclinical and clinical characteristics, rendering the evaluation of these tissue-protective agents imperative in human clinical trials. Moreover the demonstration of rhEPO and its analogues' broad neuroprotective effects in animal models of cord lesion and in human trial like stroke, should encourage scientists and clinicians to design clinical trials assessing the efficacy of these pharmacological compounds on SCI.

## 1. Introduction

Several studies published in recent years have shown that the cytokine erythropoietin (EPO) is a crucial mediator of injury-related tissue protection in mammals following ischemic and nonischemic injuries. Severe spinal cord injury (SCI) causes an immediate paralysis of muscles innervated by motoneurons caudal to the injury site. This results not only from a loss of supraspinal tracts that subserve voluntary initiation of movement, for example, corticospinal and reticulospinal tracts that use fast glutamatergic synaptic transmission, but also from a loss of descending brainstem tracts that provide spinal motoneurons with their major source of neuromodulators, such as 5-HT. From a pathophysiological perspective, SCI has historically been divided into two distinct phases. Primary (mechanical) injury directly disrupts tissues but, in the acute phase, frequently causes only limited neuronal death surrounding the lesion epicenter and damage axons and blood vessels at the site of injury, leading to vasoconstriction, hemorrhage, and ischemia [1]. As a response to primary injury, a vigorous inflammation is initiated and is followed by a cascade of secondary events such as fluid-electrolyte imbalance, regional blood flow alterations, calcium-mediated cellular injury, free-radical generation, glutamate-induced excitotoxicity, disturbances in mitochondrion function, proinflammatory cytokine production, and apoptotic cell death. This causes the attraction of inflammatory cells such as neutrophils, macrophages, and resident microglia. The consequence of this phenomenon is the amplification of injury by releasing proinflammatory cytokines [2]. During the weeks following trauma, the site of SCI is characterized by disrupted axons and a cystic cavity encased within a glial scar. Intact tissue surrounding the lesion is found in variable amounts. It is in this intact tissue that neurons are found either uninjured or with part of their myelin sheaths lost. These neurons have the potential to regenerate axons. Nonetheless, axonal regeneration frequently fails for two reasons: first, elements within the lesion environment inhibit axonal growth and second, neurons of the CNS themselves exhibit a weak intrinsic ability to regenerate axons after trauma [3, 4].

## 2. Erythropoietin Historical Background, Structure, and Signalling

A century ago, it was clear that the production of erythrocytes was modulated by a humoral factor; Carnot and DeFlandre [5] named this factor hemopoietin, and successively, in 1948, Bonsdorf and Jalavisto first used the term erythropoietin [6]. In 1977, erythropoietin was isolated from urine of anemic patients; this opened the way to the identification of both its amino acid sequence and gene [7]. These findings opened the field of recombinant human EPO, improving the quality of life of more than a million patients affected by anemia [8]. The gene encoding EPO is located in chromosome 7q11-q22, occupies a 5.4 Kb region, and contains five exons and five introns and code for a propeptide of 193 amino acids [9]. Erythropoietin is produced by all vertebrates and is an acidic glycoprotein member of the type I cytokine superfamily. The mature form of the peptide is constituted by 165 amino acids with a molecular weight of 30.4 kDa. It is a heavily glycosylated protein hormone possessing three Asn-linked sugar chains at Asn24, 38, and 83, and one mucin-type sugar chain at Serl26 [10]. These sugar chains donate 40% of the mol. wt of the peptide and probably cover most of its molecular surface. Thus, the structure of the sugar chains has increased importance in HuEPO, and it soon became obvious that producing HuEPO in a heterologous host using recombinant gene technology faced many problems. It has been shown, for example, that sialic acids affect HuEPO's *in vivo *activity by hiding the penultimate galactose residue from the asialoglycoprotein receptor [11, 12]. The EPO actions are exerted by the interaction with a specific receptor (EPO-R) which belongs to the type I family of single-transmembrane cytokine receptors [8]. The gene of EPO-R is located on chromosome 19p and contains eight exons and seven introns and encode the synthesis of a peptide with a molecular weight of 66 kDa and is constituted of 507 amino acids [13]. In physiological conditions, the EPO serum levels are very low and tissue hypoxia may cause their increase of about 50-fold. Its classical function is the regulation of erythroid development, allowing the maturation of erythroid precursors by the inhibition of apoptosis (programmed cell death) although in the last years, other important actions has bee attributed to this factor (see below). In intrauterine life of mammals, EPO is produced by the liver until late gestation, when a switch is gradually initiated from liver to kidneys [14]. In adult, this organ become the primary site of EPO production [13] although the liver contributes to the 10%–15% of EPO plasma levels. Expression of EPO mRNA has also been found in brain cortex, cerebellum, hippocampus, pituitary gland, placenta, testes, spleen, and lung [15–17]. Erythropoietin is mainly regulated in the kidney in response to hypoxia, but other factors are also involved in specialized tissues [18]. In contrast to erythropoietin, the expression of EPOR is not appreciably sensitive to hypoxia [19], rather, it is regulated by pro-inflammatory cytokines [20] such as tumour necrosis factor-*α* (TNF*α*) and IL-1*β*, erythropoietin itself [21] and probably other factors that have not yet been identified. In mammals, endogenous erythropoietin (EPO) can function either in the kidney-bone marrow system or in a local autocrine-paracrine system. In both systems, hypoxia-inducible factor (HIF) has a crucial role in regulating erythropoietin expression. In addition, erythropoietin expression in the autocrine-paracrine system can be activated through other receptor systems (e.g., insulin-like growth factor). In the kidney-bone marrow system, renal hypoxia induces the synthesis of HIF by the kidney, which, in turn, increases renal erythropoietin production. Newly synthesized erythropoietin enters the circulation and travels to the bone marrow, stimulating erythroid precursor survival and maturation. Increased oxygen delivery to tissues as a result of increased erythrocyte production attenuates the action of HIF in a negative feedback manner. The signalling pathway involves activation of Janus tyrosine kinase 2 (JAK2), which further propagates the signal by engaging secondary signalling molecules, including signal transducer and activator of transcription (STAT), Ras-mitogen-activated protein kinase (MAPK) and phosphatidylinositol 3-kinase (PI3K). In erythroid progenitor cells, this results in the upregulation of antiapoptotic proteins of the B-cell leukaemia/lymphoma 2 (BCL2) family, such as BCL-XL [22]. In addition, the activation of MAPK by JAK2 causes the activation of GSK3b leading the inhibition of caspase activation [23]. Additional EPO actions has been showed such as the modulation of intracellular calcium concentration in excitable cells (neurons and vascular smooth muscle cells) by the activation of phospholipase C-gamma (PLC*γ*) [24]. Hormonal clearance of erythropoietin from the serum occurs mainly through receptor-mediated endocytosis in the bone marrow although pharmacological doses of this cytokine are also eliminated by the liver and kidneys [25]. Since the early 1990s, it has emerged that EPO has cytoprotective effects in a wide variety of tissues, including the brain, kidney, and heart, from ischemic or nonischemic injury [26]. In its nonerythropoietic functions, EPO is produced locally by many tissues in response to physical or metabolic stress and acts in a paracrine-autocrine manner. Tissue-protective actions of EPO have been shown to be mediated by a tissue-protective receptor complex consisting of the EPO-R and the *β* common-receptor (CD131) subunit that is also used by GM-CSF, IL-3, and IL-5 [27]. Tissue injury induces metabolic stress and the release of pro-inflammatory cytokines, which activate HIF and increase local erythropoietin production. Pro-inflammatory cytokines can also directly inhibit the production of erythropoietin but greatly upregulate the expression of erythropoietin receptors (EPORs). The tissue-protective actions of erythropoietin have similarities to those activated during erythropoiesis, where JAK2, MAPK, PI3K, and NFkB all seem to be important. Activated JAK2 initiates signal transduction interacting with several adaptor factors such as Src homology containing protein (SHC), growth factors receptor-binding protein 2 (GRB2), son of sevenless protein-1 (SOS-1), and PI3K. This determine the activation of downstream messengers such as RAS, serine/threonine-specific kinase (RAF1), ERK1/2, protein kinase B (AKT) [27]. Moreover, Brines and coworkers suggested that a receptor distinct from those expressed by erythroid precursors specifically mediates tissue protection [27]. This receptor probably consists of the EPOR monomer and a dimmer of the *β* common receptor (*β*CR)—a shared receptor subunit of interleukin 3 (IL-3), IL-5 and granulocyte-macrophage colony stimulating factor (GMCSF). The exact mechanisms by which EPO exhibits its neuroprotective effect are not fully understood. Yet, as possible mechanisms have been proposed inhibition of apoptosis, modulation of nitric oxide synthesis, neurotransmitter release, and restoration of vasculature integrity [4, 28, 29]. EPO also seems to play a role in CNS in promoting neural progenitors cells and promoting ischemic preconditioning [30].

## 3. Is Erythropoietin the Right Molecule?

Ordinarily, the hematopoietic activity of EPO is distinct from its tissue-protective roles because of the large differences in concentrations required for each function, and the separate tissue compartments limit the possibility of cross-talk. For example, the affinity of EPO for the EPOR expressed by hematopoietic cells is ~100- to ~1000-fold higher than for the receptor expressed by neural cells [31]. All animal models studied so far have required high doses of rhEPO for tissue protection above those conventionally used for the treatment of anaemia, which will activate haematopoiesis. Hematopoietic activity is undesirable in the setting of injury, because increases in haematocrit (rheological abnormalities) and prothrombotic activities act in concert to reduce effective tissue perfusion. For example, an animal model with increased expression of endogenous EPO exhibits increased cerebral infarct size following arterial occlusion in spite of high levels of EPO within the brain [32]. In addition to adverse effects at the level of injury, serious systemic complications are possible, as, for example, the well-publicized fatal outcomes observed following “blood doping” by athletes [22]. Furthermore, new data reveal that many malignancies express receptors for and respond to EPO by an increased mitotic rate [33]. It is particularly worrisome that a large clinical trial evaluating the use of EPO in patients with metastatic breast cancer was recently halted after an increase in mortality within the EPO treatment arm due to tumor progression and/or thrombotic events [34]. Development of a molecule that is devoid of hematopoietic activity but is still active as a tissue protectant is theoretically possible. Although only a single gene for the EPOR has been described, EPORs obtained from tissues differ in molecular weights and affinity for EPO [31, 35]. Likewise, receptor-signalling pathways have not been completely defined but include both the Jak2 STAT5 system used in erythrocyte maturation [36] as well as the NFkB system so important for cell survival [37]. It is unclear at present whether different signalling themes are expressed by different tissues. In spite of current limitations of knowledge, promising work to define new non-EPO analogues is currently in advanced stages of development. One alternative strategy to differentiate the hematopoietic and tissue-protective activities of EPO is based on the observation that neuroprotection by EPO occurs via a gene expression program requiring only 5 minutes of exposure [38]. In contrast, during hematopoiesis, a continuous population of new cells appears, which require a continued presence of EPO. Thus, EPO with a reduced half-life will preferentially target tissue injury rather than the bone marrow. In fact, it is straightforward to reduce the serum half-life of EPO (to <2 min) by removing the sialic acid moieties terminating the oligosaccharides of the molecule. Asialo-EPO has been produced by use of sialidase, this molecule is not erythropoietic *in vivo* even up to doses of 500 ug/kg body weight in mice (equivalent to 50,000 erythropoietic units) [28]. The multiple mechanisms by which EPO is active, as well as the successful phase II trial in human stroke, suggest that the use of rhEPO will likely translate successfully into human trials. However, the hematopoietic activities of rhEPO could lead to serious hematopoietic and neoplastic complications, particularly following multiple doses. The nonhematopoietic analogues that are currently under development offer a promise of novel therapy for a wide variety of tissue injuries by specifically targeting an endogenous protective system that is a component of the innate immune response.

## 4. Erythropoietin Analogues and Related Pharmacological Properties

Nonhematopoietic EPO analogues may represent a novel class of drugs for stroke therapy. The advantage of using nonhematopoietic EPO analogues it to avoid the stimulation of hematopoiesis and thereby the prevention of an increased hematocrit with a subsequent procoagulant status or increased blood pressure [39]. 

Modified erythropoietin molecules which manifest solely tissue protective or erythropoietic activity may have a more target effect. As an example, transformation of lysine to homocitrulline by carbamylation gives rise to CEPO (Carbamylated Epo). CEPO shows only tissue-protective effects. In 2006, a study on focal cerebral ischemia rat model showed that postischaemic intravenous treatment with CEPO lead to improvement of functional recovery [40]. Mahmood and colleagues investigated the effect of intraperitoneally infused recombinant human erythropoietin (rHuEPO) and CEPO in traumatic brain injury rat model and concluded that both compounds are equally effective in enhancing spatial learning and promoting neural plasticity, but haematocrit was significantly increased only with rHuEPO [41]. Similarly, a study by Wang et al. demonstrated equivalent effects of rHuEPO and CEPO in the reduction of neurological impairment in rats subjected to embolic middle cerebral artery occlusion [42]. As expected, rHuEPO, but not CEPO, produced a transient increase in haematocrit levels [42]. Moreover, experimental results revealed that the sialic acid content of the erythropoietin molecule is directly associated with its circulating half-live and bioactivity. Darbepoietin is a rHuEPO analogue which contains more sialic acid residues. Consecutively, that molecule exerts longer circulating half-life and thereby *in vivo* potency. On the contrary, asialic EPO is generated by enzymatic desialylation of rHuEPO and manifests attenuated *in vivo *erythropoietic activity and shorter half-life (minutes); however, it retains its neuroprotective effects [28, 43].

In addition to the development of structurally related EPO derivatives or mimetics (structural variants) that target EPOR, compounds that acts by induction of EPO gene expression (functional EPO variants), such as HIF-stabilizers [44–46] and GATA-2 inhibitors [47] are actively pursued as alternatives to EPO for stimulation of erythropoiesis and ultimately also for neuroprotection. The advantages and disadvantages of agents that ubiquitously induce a wide spectrum of hypoxia inducible genes are presently not clear. In particular, the question whether these agents have benefits over the use of rHuEPO in the treatment of nervous system disorders remains to be addressed, except for the role of hypoxia inducible genes activation on adult neural stem cells, where that process results on EPO production and increase of cell survival and differentiation in mature neurons reaching a rate of 40% after prolonged ischemia as demonstrated in our recent studies [48].

The intracerebral injection of recombinant human erythropoietin in either transient forebrain ischemia [49–51] or regional brain ischemia [52] causes neuroprotection. EPO was administered via a transcranial route in prior studies of brain ischemia because EPO does not cross the blood-brain barrier (BBB). The lack of EPO penetration of the brain following intravenous (IV) administration has been demonstrated in both the mouse [53] and the Rhesus monkey [54]. The poor BBB penetration of EPO from blood could, at least in part, explain the failure to produce neuroprotection in human stroke following treatment with i.v. EPO in the first 6 h following the infarction [55]. Thus, the development of EPO as a drug for CNS disorders such as stroke requires that the neurotrophin be re-engineered to cross the BBB. This is possible by genetic fusion of EPO to a BBB molecular Trojan horse. The Trojan horse is an endogenous peptide, or peptidomimetic monoclonal antibody (MAb), that crosses the BBB via receptor-mediated transport. Recently, human EPO was re-engineered as an IgG-EPO fusion protein, where EPO was fused to a genetically engineered MAb against the human insulin receptor (HIR) [54]. This study show that HIRMAb cross-reacts with the insulin receptor in the Rhesus monkey, and the HIRMAb-EPO fusion protein was shown to rapidly penetrate the BBB in the Rhesus monkey following IV injection. The potent neuroprotection property of the HIRMAb-EPO fusion protein was investigated in a rat stroke model, the permanent middle cerebral artery occlusion (MCAO) model [56]. Following the intracerebral injection of picomole doses of HIRMAb-EPO fusion protein, the stroke volume was reduced 98% in the rat [56]. The HIRMAb-EPO fusion protein was injected directly into the brain in the rat MCAO model, because the HIRMAb part of the fusion protein does not recognize the rat insulin receptor [57]. There is no known MAb against the rat or mouse insulin receptor that could be used as a BBB Trojan horse in rodents. For that reason, it has been proposed the delivery of neurotrophins such as EPO across the mouse BBB is possible with a surrogate Trojan horse, which is a genetically engineered MAb against the mouse transferring receptor (TfR), which is designated the cTfRMAb [58]. The cTfRMAb is comprised of variable regions from an original rat IgG, which are fused to mouse heavy chain and light chain constant regions, and is specific for the mouse TfR with no reactivity for the rat TfR. Recently, a fusion protein of the cTfRMAb and EPO, designated the cTfRMAb-EPO fusion protein, has been engineered and expressed [59]. Fusion protein rapidly crosses the BBB in the mouse, and the brain uptake following i.v. injection is 2.0 ± 0.1% of injected dose (ID)/gram [59]. The cTfRMAb-EPO fusion protein retains high affinity binding for the mouse EPO receptor (EPOR) with a KD of 0.33 ± 0.04 nM [59]. Therefore, the purpose of the present investigation was to examine the neuroprotective properties of i.v. administration of the cTfRMAb-EPO fusion protein in a permanent MCAO model in the mouse. Recombinant EPO was also administered i.v. in this study. MCAO study in the mouse provides the basis for future work in MCAO models in higher animals, such as the primate, using the HIRMAb-EPO fusion protein [54]. The study presented by Zhou and colleagues [60] shows that an 81% reduction in stroke volume is achieved with a brain IgG-EPO concentration of 600 ng/g. The brain uptake of the HIRMAb-EPO fusion protein in the Rhesus monkey is 2.1 ± 0.1% ID/100 g brain [54]. 

## 5. Preclinical Studies

Erythropoietin and EPO-R have been documented to play important roles in SCI. This is the case for both endogenous and exogenously administered EPO [14]. Of great clinical importance is also the expression of EPO after SCI which has been shown to be a part of the physiological response to hypoxia. Bernaudin and colleagues [52] reported that in the encephalic areas surrounding ischemic brain lesions, cells increased the expression levels of EPO and its receptor mRNAs. In particular, the upregulation of EPO receptor occurred first in neurons and endothelial cells of the microcirculation and was followed by an increase in EPO expression by astrocytes and neurons. This increase of EPO has been proposed as a mechanisms that, by apoptosis inhibition, could reduce the inflammatory response, and thus a reduction of secondary injury in rats [61]. The work of many groups demonstrated that the administration of exogenous rhEPO in animal models (rats) of traumatic SCI produces substantial neuroprotection [43, 62–70]. Gorio et al. [62] and coworkers in two different models of traumatic spinal cord lesion (transient compression or blunt trauma) showed that the single dose i.p. administration of rhuEPO gives a markedly superior clinical course of recovery of motor function compared with placebo, characterized by an earlier and more complete normalization of function over a 28-day period of study. Moreover, the same authors observed that secondary inflammation was also markedly attenuated by rhEPO administration and associated with reduced cavitation within the cord. These results suggested that beneficial effect of rhEPO treatment occurs within the first week after injury. 

The exact mechanisms by which EPO exhibits its neuroprotective effects are still under investigation. In particular, exogenous EPO administrations leads to improvement in cognitive outcome as well as in (sensory-) motor functions, together with a diminution of lesion volume, brain oedema, inflammation, and apoptosis. Recently, Huang and coworkers in rat contusion model of SCI showed that the administration of a single dose of rhEPO immediately after cord injury improved motor function recovery, decreased lesion severity, and increased neuronal regeneration. This work evidenced that the protective effect of exogenous EPO was mediated by the increase of MKP-1 (Mitogen Kinase Phosphatese-1) expression and the decrease of MAP kinase activity (p-ERK1/2). The chronic effects mediated by a single exposure of rhuEpo were investigated in a contusion model of SCI in rat by Vitellaro-Zuccarello and coworkers [70]. The work of these researchers showed that rhEPO administration after SCI modifies astrocytic response to injury by increasing AQP4 (Aquaporin-4) immunoreactivity in the spinal cord, but not in the brain, without apparent modifications of dystrophin and syntrophin distribution. They also observed an attenuation of astrogliosis, and a significant increase of the relative volume of a microvessel fraction, indicating a proangiogen or a vasodilatory effect of rhEPO. However, it has been reported that a genetic reduction in reactive astrocytes in a mouse model has been associated with a worse clinical outcome after a stab injury to the spinal cord [71]. Thus, the meaning of a reduced astrogliosis caused by EPO needs further investigations. Taken together, the available data on Erythropoietin's beneficial effects in animal models of SCI are due to limitation of damage following injury and enhancement of neuronal regeneration. Neural stem cells present in SC proliferate to form spheres of undifferentiated cells that produce neurons, astrocytes, and oligodendrocytes. Cultured stem cells when exposed to EPO produced two to threefold more neurons [29, 48]. Thus, EPO might contribute to recovery after SCI by increasing the number of new neurons. 

## 6. Human Trials

Taken collectively, the evidences presented on the preclinical evaluation studies of EPO effects on spinal cord injury, it is reasonable to assume that exogenous and endogenous EPO and EPO-R system acts as a protective mechanism that becomes rapidly activated after injury to promote neuronal survival. Published clinical studies on EPO in neurological and psychiatric indications are still rare even though many studies are ongoing worldwide. Concerning the administration of exogenous EPO on spinal cord injury, only two trial were found on databases [72], one of them is an Italian multicenter study and concerns the evaluation of tolerability and efficacy of erythropoietin (EPO) treatment in spinal shock: comparative study versus methylprednisolone (MP). Primary objective of the study is to assess the superiority of EPO compared to MP in improving the clinical outcome of SCI (ASIA impairment scale); secondary objectives are: to assess the safety of EPO compared to MP, the effects on the motor and sensory functions and on improving functional autonomy, the influence on spasticity and neurogenic pain, and, the impact on surrogate end-points (Somatosensory Evoked Potentials and Magnetic Resonance Imaging). The second study was conducted in Canada and dedicated to the treatment of patients with malignant spinal cord compression (MSCC) who are paraparetic or paraplegic before initiating treatment, the current treatment options provide a meagre-to-poor chance of neurologic recovery, and the prognosis is guarded. Improving the chance of ambulation after treatment for MSCC may dramatically improve patients' quality of life, decrease days spent in hospital, and improve survival. Steroids appear to prevent neurologic damage from MSCC and increasing doses appear to have an increasingly protective effect; however, higher doses are limited by an increasing incidence of serious toxicity [72].

## 7. Erythropoietin and Neural Stem Cells

Studies of neural stem and progenitor cells play a very important role to understand the mechanisms of differentiation of the cells into lineage specific cells like neurons and astroglia. Several studies have shown that neurogenesis is enhanced after hypoxia and that erythropoietin (EPO) is produced in the brain as part of the intrinsic hypoxia response. Thus, Shingo and coworkers investigated the effects of exogenous EPO on *in vitro* culture of stem cells. They showed that exogenous EPO acts directly on cultured embryonic NSCs, promoting the production of neuronal progenitors [29], suggesting a possible role of EPO in neural stem cells differentiation. Carbamylated erythropoietin (CEPO), a well-characterized erythropoietin (EPO) derivative, does not bind to the classical EPO receptor and does not stimulate erythropoiesis. Wang et al. found that CEPO significantly increased adult neural progenitor cell proliferation and promoted neural progenitor cell differentiation into neurons, which was associated with upregulation of Sonic hedgehog (Shh), its receptor ptc, and mammalian achaete-scute homolog 1 (Mash1), a pro-neuron basic helix-loop-helix protein transcription factor [73]. The same authors by using a coculture system of mouse brain endothelial cells and neural progenitor cells derived from the subventricular zone of adult mouse, investigated the hypothesis that neural progenitor cells treated with rhEPO promote angiogenesis. Their *in vitro* results suggested that EPO enhances VEGF secretion in neural progenitor cells through activation of the PI3K/Akt and ERK1/2 signalling pathways and that neural progenitor cells treated with rhEPO upregulated VEGFR2 expression in cerebral endothelial cells, which along with VEGF secreted by neural progenitor cells promoted angiogenesis [74]. Giese et al. demonstrated that differentiation of human foetal neural progenitor cells under hypoxic conditions results in an increased neurogenesis. Moreover, their expansion and proliferation under lowered oxygen conditions also increased neuronal differentiation, although proliferation rates were not altered compared to normoxic conditions. Erythropoietin partially mimicked these hypoxic effects, promoting an increase of the metabolic activity during differentiation and exerting protection of differentiated cells from apoptosis [75]. Recently, we directed our efforts to the isolation of neural precursor cells (NPCs) capable of resisting to a prolonged ischemic insult, as this may occur at the site of traumatic and ischemic CNS injuries. Adult neural precursors from mice postmortem brain were isolated, grown *in vitro*, and their differentiation capability was investigated by evaluating the expression of different neuronal markers. This new type of neural stem cells were called postmortem neural precursors (PM-NPCs). Under differentiation conditions, our PM-NPCs yield mostly neurons (about 30%–40%), show activation of hypoxia-inducible factor-1 and MAPK, and express both erythropoietin (EPO) and its receptor (EPO-R). The exposure of PM-NPCs to neutralizing antibodies to EPO or EPO-R dramatically reduced the extent of neuronal differentiation to about 11% of total PM-NPCs. The functionality of mTOR and MAPK is also required for the expression of the neuronal phenotype by PM-NPCs. These results suggest that PM-NPCs can be isolated from animal cadaver even several hours after death and their self-renewable capability is comparable to normal neural precursors. Differently, their ability to achieve a neural phenotype is superior to that of NPCs, and this is mediated by the activation of hypoxia-induced factor 1 and EPO signaling. PM-NPCs may represent good candidates for transplantation studies in animal models of neurodegenerative diseases [48].

## 8. Conclusions

Recently, research has focused on rhEPO and its non-erythropoietic derivatives investigating their effects on SCI treatment as well as the molecular mechanisms involved such as antiapoptotic, anti-inflammatory functions, oedema reduction leading to neuronal and oligodendrocytes survival [36], and restoration of vascular integrity. Moreover, researchers suggested a contribution of EPO to neurons regeneration. The remarkable safety profile of rhEPO therapy in anemia and the demonstration of rhEPO and its analogues' broad neuroprotective effects in animal models should encourage the design of clinical trials to assess the efficacy of therapy of these proteins on SCI. Clinical evaluation end points should include besides quality of life assessment, motor, and sensory and autonomic function. 
